# Analysis on carbon emissions efficiency differences and optimization evolution of China’s industrial system: An input-output analysis

**DOI:** 10.1371/journal.pone.0258147

**Published:** 2022-03-24

**Authors:** Shuang Wang, Yang Yu, Tangyang Jiang, Jun Nie

**Affiliations:** 1 School of Public Affairs, Chongqing University, Chongqing, China; 2 School of Economics, Hainan University, Haikou, Hainan, China; 3 China Center for Competition Policy, Haikou, Hainan, China; 4 School of Internet, Anhui University, Anhui, China; Institute for Advanced Sustainability Studies, GERMANY

## Abstract

The excessive carbon emissions not only intensify the global climate change, but also seriously restrict the sustainable development of social economy. However, improving industrial carbon emissions efficiency is the most directly effective way to reduce emissions. Therefore, accurate measurement and analysis of carbon emissions efficiency and evolution characteristics of China’s industrial system is the basis for China to improve carbon emissions efficiency. Based on this, we adopted energy consumption method and input-output method to calculate and analyze the industrial carbon emissions efficiency and evolution characteristics of China from 2002 to 2015. The results show that (1) If carbon emissions from cement production are ignored and only energy-related carbon emissions are considered, the calculation results of carbon emissions efficiency of heavy industry will be overestimated about 30%. (2) Compared with 2002, China’s industrial carbon emissions efficiency increased by about twice in 2015. Specifically, the optimization of carbon emissions efficiency in agriculture, construction, light industry and service industry is very obvious, which has increased by 5.65, 5.00, 4.69 and 4.68 times respectively; The optimization of carbon emissions efficiency in power sector, heavy industry, transportation and chemical industry was obvious, which increased by 2.55, 2.01, 1.86 and 1.47 times respectively; The carbon emissions efficiency of the fossil energy sector has decreased by 0.36 times. (3) The carbon emissions efficiency of various industries has significant differences. Among them, agriculture, service industry, light industry and construction industry are generally industries with higher carbon emissions efficiency, while power sector, fossil energy sector, transportation industry, chemical industry and heavy industry are generally industries with lower carbon emissions efficiency, especially power and fossil energy sectors. Based on these, this paper provides policy implications and scientific evidence for accurately improving carbon emissions efficiency from the perspective of carbon emissions efficiency.

## Introduction

At present, the climate change problem caused by the greenhouse effect is attracting a lot of attentions around the world [1]. The global carbon project conducted a preliminary calculation of global carbon emissions, and found that the carbon emissions increased by about 2% in 2018. This is another growth in global carbon emissions after a small rise of 1.4 percent in 2017. Significantly, China accounted for 27% of global emissions, and its carbon emissions increased by about 4.5% during the same period. Surprisingly after several years of decline, America also contributed 2.5 percent growth accounting for 15% of global emissions. It reaches a consensus that controlling excessive carbon dioxide emissions has become the most effective way to protect the ecological environment and mitigate the greenhouse effect.

To reduce carbon emissions, countries around the world actively carry out cooperation. At the Copenhagen climate conference in 2009, Chinese government officially announced that China’s carbon emissions per unit of GDP would reduce by 40% to 45% by 2020 compared with 2005. At the Asia-Pacific Economic Cooperation (APEC) meeting in 2014, Chinese government pledged that China’s carbon emissions would peak by 2030 and work hard to reach the peak before then. The aforesaid commitments indicate that it is the endeavor of Chinese government to mitigate global warming. Therefore, it is essential to analyze the sources of China’s carbon emissions. They include energy consumption, cement production process and biomass decomposition [2–4], etc. According to the Intergovernmental Panel on Climate Change (IPCC), most of China’s carbon emissions come from energy consumption. Moreover, due to the large scale of China’s construction industry, carbon emissions from cement production also account for a large proportion of total carbon emissions. Other carbon emissions sources, such as biomass decomposition, are not the focus of emission reduction due to their small share.

Since China is still a developing country, it is also important to protect the environment while maintaining economic growth. To balance economic development and carbon emissions, a series of energy-saving and emissions reduction measures have been formulated by various countries so as to alleviate the dilemma between economic development and environmental protection. From the practices of the developed and under-developed economies, there are mainly three measures for carbon emissions reduction, namely optimizing the energy consumption structure, developing renewable energy technology and developing carbon trading market. Some scholars studied the impact of optimizing the consumption energy structure on the energy efficiency. Their results affirmed the positive effect of optimizing the consumption energy structure on energy conservation and emissions reduction [5–7]. As for the development of renewable energy technology, the renewable energy system restricted by the economic development level plays a key role in developed countries [8]. Renewable energy policy may have a positive or negative impact on the implementation of renewable energy system [9]. However, under the framework of the Kyoto protocol, carbon trading market emerged as an important measure of energy conservation and emissions reduction [10]. After the establishment of the international exchanges, namely European Union Greenhouse Gas Emission Trading Scheme, UK Emissions Trading Group, Chicago Climate Exchange of the United States, and Australian National Trust, have gradually developed carbon trading markets. Carbon trading can not only promote enterprises’ independent innovation, but also coordinate capital operation through the market mechanism, so as to effectively reduce carbon emissions [11].

For a long time, the staged rigid demand for energy determines that energy production and consumption structures are difficult to change in the short term in China. Therefore, optimizing China’s energy consumption structure to solve the pressure of emission reduction is an important task. Although most scholars argue that renewable energy development is conducive to the economic growth, few scholars raised objections [12]. These uncertainties have limited the development of renewable energy to some extent. Meanwhile, due to the late establishment, immature development and inactive trading of China’s carbon trading market [13], the contribution to intermediate emission reduction is limited. Therefore, it can be concluded that through these three ways to effectively carry out emissions reduction in a short time, China is facing great pressure.

However, we can improve the carbon emissions efficiency through technology upgrades and management innovation, and effectively reduce the carbon emission in a short term to promote stable economic development. Therefore, it is both of theoretical and practical significance to carry out a study on the difference and optimization degree of carbon emissions efficiency in China’s industrial system. This study not only examines the differences in carbon emissions efficiency of various industries in the industrial system from 2002 to 2015, but also dynamically indicates the optimization degree of carbon emissions efficiency of various industries during the period. Moreover, the study provides guidance for carrying out emissions reduction from the perspective of the carbon emissions efficiency of various industries within the actual affordability of the Chinese government in the future.

## Literature review

We have combed and summarized a lot of domestic and foreign research literature on carbon emissions efficiency. At the theoretical level, carbon emissions efficiency refers to the ratio between the carbon emissions and the output, which has attracted attentions from a large number of scholars [14–17]. It is found that carbon emissions efficiency can be roughly divided into two categories, that is, industrial carbon emission efficiency and regional carbon emission efficiency.

### Industrial carbon emissions efficiency

The industrial carbon efficiency has been analyzed by many researchers for one or several industries. Some scholars studied the carbon emissions efficiency and energy efficiency of industrial sectors, which is of guiding significance for the government to formulate industrial policies [18, 19]. By analyzing the carbon emissions efficiency of the metallurgical industry, Wu et al., (2016) [20] pointed out that the adoption of industrial symbiosis measures (ISM) can improve the overall efficiency and energy efficiency. Lin and Xu (2018) [21] found that technological progress is the main factor to improve efficiency. Pardo et al. (2011) [22] analyzed the possibility of improving energy efficiency and reducing carbon emissions in the cement industry by 2030. Their results showed that thermal energy consumption would significantly be reduced in 2030. Griffin (2017) [23] examined the carbon emissions efficiency of British chemical industry, and analyzed the uncertain impact of key technologies and non-technical barriers on the improvement of carbon emissions efficiency. Zhou et al. (2020) [24] adopted super slacks-based measure (SBM) model to calculate carbon emissions efficiency, and further analyzed the coupling degree between carbon emissions efficiency and industrial structure upgrading. It was found that obvious dynamic imbalance existed between carbon emissions efficiency and industrial structure upgrading in China with the ‘low level trap’ of regional carbon emissions efficiency being more serious than that of regional industrial structure upgrading. Gao et al. (2021) [25] measured the embodied carbon emissions of 28 industries in China from 2005 to 2017, and further analyzed the carbon emissions efficiency of 28 industries under embodied carbon emission and direct carbon emission scenarios. Their research results showed that direct carbon emissions and the overall efficiency of carbon emissions in China’s industries had improved, accompanied by significant heterogeneity among different industries. In addition, indirect carbon emissions efficiency is lower than direct carbon emissions efficiency.

### Regional carbon emissions efficiency

From a regional perspective, Xin et al., (2015) [26] studied the energy efficiency, carbon emissions performance and carbon emissions reduction potential of different regions in China. They pointed out that there was significant regional heterogeneity in energy conservation and emission reduction in China, and proved that the conclusion drawn by ignoring regional heterogeneity is biased. Meng et al., (2016) [27] evaluated regional energy efficiency and carbon emissions efficiency in China’s 30 provinces during 1995–2012. The results indicated that China’s energy efficiency and carbon emissions efficiency were stable during the ninth five-year plan, and declined during the 10th five-year plan. However, during the 11th five-year plan, the eastern region showed highest efficiency, while the central region exhibited the lowest efficiency. Iftikhar et al., (2018) [28] studied the energy efficiency and carbon emissions efficiency at the national level, and found that the larger economies with production intensive strategies, more secondary industries and weaker carbon tax laws were more likely to be less efficient, indicating that India, China and Russia has good potentials to improve efficiency. Some scholars simultaneously examined the carbon emissions efficiency of industries and regions. For example, Wang and Wei (2014) [29] analyzed the energy efficiency and carbon emissions efficiency of China’s industrial sectors, and found that coastal areas with rapid economic development have higher efficiency. Andersson et al. (2018) [30] analyzed the impact of corporate ownership structure on carbon emissions from the perspective of provincial data in China. Their results showed that the carbon emissions of private enterprises were less than that of state-owned enterprises. Wang et al., (2019) [31] evaluated the carbon emissions efficiency of three industries in different regions of China. They found that the efficiency in eastern region was higher than that in central and western regions, and the tertiary industry was higher than the primary and secondary industries. Xu et al. (2018) [32] proposed a competition mechanism for carbon emissions allocation to achieve the coordination between economic environment and sustainable development by adopting the dynamic planning method, which can be applied to different countries and industries by slightly adjusting some parameters. Zhang et al. (2020) [33] evaluated the impact of China’s emission trading system on carbon emissions reduction, and found that the emission trading policy implemented in the pilot areas significantly reduced carbon emissions, especially in the economically developed areas of eastern China. Wang and Zheng (2021) [34] studied the spatial effects and influencing factors of carbon emission intensity in 30 provinces of China from 2005 to 2017. Their results showed that from 2005 to 2017, China’s carbon emission intensity gradually decreased from east to west and from south to north. In addition, reducing energy intensity can reduce carbon emissions intensity to a great extent.

The above literature analyzed carbon emissions efficiency from industrial and regional perspectives. Especially from the perspective of industry, there are two limitations in research on the carbon emissions efficiency. On the one hand, most existing literature only study the carbon emissions efficiency of a single industry, and lacks the comparison and difference analysis of carbon emissions efficiency between different industries from the perspective of the entire industry system. On the other hand, the existing literature lacks dynamic research on the evolution of the optimization degree of carbon emissions efficiency of China’s industrial system. These deficiencies led to the lack of accurate practical guidance and scientific basis when Chinese government formulated specific industrial system emissions reduction policies for the industrial system.

### The academic contribution of this paper

In view of this, the academic contribution of this paper is reflected in the following three aspects. (1) The influence of energy capital deposit and loan, energy export and carbon emission of cement production is considered in the calculation of carbon emissions efficiency. (2) From the entire industrial system, this paper dynamically analyzes the differences in carbon emissions efficiency of various industrial systems in China during different periods. (3) Finally, an attempt is made to demonstrate the optimization of carbon efficiency for each specific industry in China’s industrial system from 2002 to 2015.

## Model and data

In the present work, CO_2_ emission mainly includes two parts. One part is the CO_2_ emission from energy combustion, and the other part is the CO_2_ emission from cement production. The CO_2_ emission from energy combustion is generated by the industries. Thus, we can use the energy consumption method and input-output analysis to calculate the industrial CO_2_ emission. The second part refers to the CO_2_ emission from cement production, which can be calculated by China’s total output and related emission factors.

The excessive CO_2_ emissions not only intensify the global climate change, but also seriously restrict the sustainable development of social economy. However, improving industrial carbon emissions efficiency is the most directly effective way to reduce emissions. Therefore, accurate measurement and analysis of carbon emissions efficiency and evolution characteristics of China’s industrial system is the basis for China to improve carbon emissions efficiency. Based on this, this paper calculates and analyzes the industrial carbon emissions efficiency and evolution characteristics of China from 2002 to 2015 by using energy consumption method and input-output method.

### Method and calculation

#### The CO_2_ emissions from energy consumption

The energy consumption method is prevalent for calculating the energy-related CO2 emissions; The CO_2_ emissions from various industries are mainly derived by decomposing the total energy CO_2_ emissions according to the proportion of energy consumption in the industry. When the amount of energy combustion and the CO_2_ emissions coefficients are known, the energy-related carbon emissions are the product of the amount of energy combustion and the carbon emissions coefficient.

E=C×W (1)

Where, *E* is the CO_2_ emissions from energy combustion; *C* is the amount of energy combustion; *W* is the CO_2_ emissions coefficient. Due to the wide variety of energy sources (17 kinds of energy sources are listed in China’s Energy Balance Sheet for 2002 and 2007, while 20 kinds of energy sources are listed in China’s Energy Balance Sheet for 2012 and 2015), the total carbon emissions from energy combustion should be equal to the sum of CO_2_ emissions from the combustion of each energy source *i*, which can be calculated as follows.

$$E=\sum_{i}E_{i}=\sum_{i}C_{i}\times W_{i}$$ (2)

Where, *E*_*i*_ is the amount of CO_2_ emissions from the combustion of energy source *i*; *C*_*i*_ is the amount of energy source *i* combustion; *W*_*i*_ is the CO_2_ emissions coefficient of energy source *i*.

One of the most critical issues in the calculation of energy-related carbon emissions is how to determine accurately the total amount of energy combustion, because not all the energy sources are used for combustion. Due to data availability from China’s energy statistical yearbook, we can determine the amount of energy combustion as follows.

$$C_{i}=C_{i}^{T}+C_{i}^{P}+C_{i}^{H}-C_{i}^{M}$$ (3)

Where, $C_{i}^{T}$ is the amount of energy source *i* combustion for final consumption;$C_{i}^{P}$ is the amount of energy source *i* combustion for thermal power production; $C_{i}^{H}$ is the amount of energy *i* combustion for heating production; $C_{i}^{M}$ is the amount of energy *i* for the industrial raw materials. The results obtained by using the Eq (3) are shown in Tables 1 and 2.

**Table 1:** Energy use of China in 2002 and 2007.

Categories of energy	Flaming energy in 2002(10^4^t)	Flaming energy in 2007(10^4^t)
Raw Coal	108964.07	193588.17
Cleaned Coal	1190.55	2101.38
Other Washed Coal	3886.24	9520.79
Briquettes	835.7	1133.19
Coke	11405.14	28876.06
Coke Oven Gas(10^8^m^3^)	263.89	569.21
Other Gas(10^8^m^3^)	607.15	954.93
Other Coking Products	195	462.68
Crude Oil	660.22	869.74
Gasoline	3740.7	5500.7
Kerosene	916.57	1238.44
Diesel Oil	7641.15	12464.84
Fuel Oil	3739.09	3956.6
LPG	1601.63	2273.83
Refinery Gas	658.71	1021.1
Other Petroleum Products	64.13	4151.5
Natural Gas(10^8^m^3^)	225.51	579.82

Note: The data is obtained from China Energy Statistics Yearbook in 2003 and 2008 and compiled by the authors.

**Table 2:** Energy use of China in 2012 and 2015.

Categories of energy	Flaming energy in 2012(10^4^t)	Flaming energy in 2015(10^4^t)
Raw Coal	266877.05	285687.12
Cleaned Coal	2671.24	5278.46
Other Washed Coal	10855.69	13287.05
Briquettes	1045.54	1905.21
Coke	37749.95	42311.07
Coke Oven Gas(10^8^m^3^)	792.79	804.46
Blast Furnace Gas(10^8^m^3^)	2431.16	7608.53
Converter Gas(10^8^m^3^)	403.71	515.76
Other Gas(10^8^m^3^)	142.07	156.4
Other Coking Products	786.72	684.56
Crude Oil	493.83	666.18
Gasoline	8122.23	11358.82
Kerosene	1956.6	2661.77
Diesel Oil	16938.68	17281.52
Fuel Oil	2303.27	2188.64
LPG	2269.6	3347.84
Refinery Gas	1474.99	1723.94
Other Petroleum Products	2312.11	2048.26
Natural Gas(10^8^m^3^)	1094.85	1466.01
LNG	1255.12	2287.49

Note: The data is obtained from China Energy Statistics Yearbook in 2013 and 2016 and compiled by the authors.

The carbon emissions coefficient of energy can be obtained by multiplying average low calorific value of energy and the CO_2_ emissions coefficient per unit heat.

$$W_{i}=T_{i}\times Q_{i}$$ (4)

Where, *T*_*i*_ is the CO_2_ emissions coefficient per unit heat of energy *i*; *Q*_*i*_ is the average low calorific value of energy *i*; *W*_*i*_ is the CO_2_ emissions coefficient of energy *i*. Table 3 shows the results obtained by using the above equation.

**Table 3:** The CO_2_ emissions coefficients of different energy sources.

Categories of energy	CO_2_ emissions per heat(kg/TJ)	The average low calorific value(10^6^J/t)	CO_2_ emissions coefficient (t/t)
Raw Coal	97967	20908	2.4083
Cleaned Coal	97967	26344	2.5808
Other Washed Coal	97967	8363	0.8193
Briquettes	97500	8363	0.8154
Coke	107000	28435	3.0425
Coke Oven Gas	44400	16726(10^3^J/m^3^)	7.4263(10^-4^t/m^3^)
Blast Furnace Gas	260000	5227(10^3^J/m^3^)	13.5902(10^-4^t/m^3^)
Converter Gas	260000	5227(10^3^J/m^3^)	13.5902(10^-4^t/m^3^)
Other Gas	260000	5227(10^3^J/m^3^)	13.5902(10^-4^t/m^3^)
Other Coking Products	97500	33453	3.2617
Crude Oil	73300	41816	3.0651
Gasoline	70000	43070	3.0149
Kerosene	71900	43070	3.0967
Diesel Oil	74100	42652	3.1605
Fuel Oil	77400	41816	3.2366
LPG	63100	50179	3.1663
Refinery Gas	57600	46055	2.6528
Other Petroleum Products	73300	41816	3.0651
Natural Gas	56100	38931(10^3^J/m^3^)	21.8403(10^-4^t/m^3^)
LNG	56100	54071	3.0334

According to the above equations, we can get the total CO_2_ from energy combustion in 2002, 2007, 2012 and 2015. In China’s input-output table, there are 42 sectors, among which four sectors are the energy sector. The relationship between the various energy sources and the energy sectors is shown in Table 4.

**Table 4:** The relationship between the various energy sources and the energy sectors.

Energy sectors	The coal mining industry	The petroleum and natural gas exploitation industry	The petroleum, cooking and nuclear fuel products industry	The gas production and supply industry
Categories of energy	The raw coal, clean coal and other washed coal	The crude oil and natural gas	The gasoline, kerosene, diesel, fuel oil, liquefied petroleum gas, refinery dry gas, coke and other cooking or petroleum sources	The coke oven gas and other types of gas

When we use the input-output analysis to calculate the China’s industrial carbon emissions, we should pay attention to the exclusion of energy capital deposits and loans and energy exports. This is because the energy capital deposits and loans are in the form of physical objects and do not produce carbon emissions, while the carbon emissions generated by energy export are in other places. Thus, these two parts must be eliminated. In this paper, we discuss the case where these two parts are eliminated or not. Firstly, without eliminating these two parts of energy consumption, the total energy investment of all energy industries for the combustion is as follows.

$$D_{j}=DI_{j}+DF_{j}$$ (5)

Where, *D*_*j*_ is the total energy investment of energy industry *j* for combustion; *DI*_*j*_ is the intermediate demand investment of energy industry *j* for combustion; *DF*_*j*_ is the final demand investment of energy industry *j* for combustion. If the two parts are eliminated, *D*_*j*_ can be computed as follows.

$$D_{j}=DI_{j}+DF_{j}-DC_{j}-DE_{j}$$ (6)

Where, *DC*_*j*_ is the fixed capital investment of energy industry *j* for combustion; *DE*_*j*_ is the exported or transferred investment of energy industry *j* for combustion. Then, the CO_2_ emissions coefficient of energy industry *j* is as follows.

$$e_{j}=\frac{E_{j}}{D_{j}}$$ (7)

Where, *e*_*j*_ is the CO_2_ emissions coefficient for combustion of energy industry *j*; *E*_*j*_ is the CO_2_ emissions for combustion of energy industry *j*. Based on the above Eq (7), the individual CO_2_ emissions for energy combustion of the 42 sectors listed the input-output table can be computed. Utilizing the Eq (8), we can measure the energy sector *j* derived CO_2_ emissions which is generated from the industry *k*.

$$E^{k}=\sum_{j}E_{j}^{k}=\sum_{j}C_{j}^{k}\times e_{j}$$ (8)

Where, *E*^k^ is the CO_2_ emissions of sector *k*; $E_{j}^{k}$ is the energy sector *j* derived CO_2_ emissions which is generated from the sector *k*; *$C_{j}^{k}$* is the energy sector *j* generated energy investment which is used for the sector *k*. Using the above process, we can calculate the individual CO_2_ emissions of 42 sectors from the energy combustion. Then we divided the CO_2_ emissions of sector *k* by the output of sector *k*. The carbon emissions efficiency of sector *k* is calculated as follows.

$$f^{k}=\frac{E^{k}}{T^{k}}$$ (9)

Where, *T*^*k*^ is the output of sector *k*; *f*^*k*^ is the CO_2_ emissions efficiency of sector *k*. In this study, we consolidated 42 sectors into 9 sectors. For details of the merging, please see S1 Appendix. However, the calculated carbon emissions efficiency in this way does not take into account the price output value conversion caused by inflation and other factors. Therefore, it is necessary to deal with the price conversion coefficient over the years. Based on this, we use the price in 2002 as the benchmark price to calculate the annual carbon emissions efficiency. The calculation formula is shown as follows.

$$P_{i}^{k}=f_{i}^{k}*T_{i}$$ (10)

Where, $P_{i}^{k}$ is the carbon emissions efficiency of sector *k* with the price conversion processing in the *i* year; $f_{i}^{k}$ is the carbon emissions efficiency of sector *k* without the price conversion processing in the *i* year; *T*_*i*_ is the price conversion coefficient in the *i* year. The price conversion coefficient from 2002 to 2015 is obtained from data collection and conversion. The detailed results are shown in Table 5.

**Table 5:** The price conversion coefficient from 2002 to 2015.

Year	Price index
2002	1
2003	1.023
2004	1.0854
2005	1.1386
2006	1.1727
2007	1.2091
2008	1.2925
2009	1.2227
2010	1.29
2011	1.3674
2012	1.3441
2013	1.3186
2014	1.2935
2015	1.2263

Note: Due to a large amount of data, only 4 digits after the decimal point were included.

#### The CO_2_ emissions from the cement production

Many chemical reactions that produce carbon emissions occur in cement production. China’s construction industry is so large that carbon emissions from this industry cannot be ignored. As the cement industry belongs to the heavy industry, the carbon emission of subsequent cement production should belong to the category of carbon emissions from heavy industry. In this paper, the equation for calculating CO_2_ emissions generated from the cement production is shown as follows.

EC=QC×u (11)

Where, *EC* is the CO_2_ emission from the cement production; *QC* is the China’s output of the cement production; *u* is the CO_2_ emission coefficient for the cement production. In this study, the CO_2_ emission coefficient for cement production is the coefficient for Portland cement which is obtained from the Greenhouse Gas Protocol. The CO_2_ emission coefficient value is 0.5021016, which implies that the CO_2_ emission yielded per ton of cement output is 0.5021016 tons. According to the China cement network, the cement output of each province in China in 2002, 2007, 2012 and 2015 can be obtained. Then, the annual cement output of each province can be summed to obtain the total cement output of China in corresponding year. The detailed results are shown in Table 6.

**Table 6:** The output of cement production from 2002–2015 in China.

Year	2002	2007	2012	2015
Cement production (hundred million tons)	7.25	13.61	21.84	23.50

Note: Due to large amount of data, only 2 digits after the decimal point were included.

### Data

The data of this paper mainly includes China’s energy balance and input-output tables for 2002, 2007, 2012, and 2015. The carbon emissions coefficient of each energy source under unit heat production is taken from the 2006 IPCC Guidelines for National Greenhouse Gas Inventories published by the global environmental strategy institute.

## Results and analysis

### The calculation of total carbon emissions

**Fig 1:**
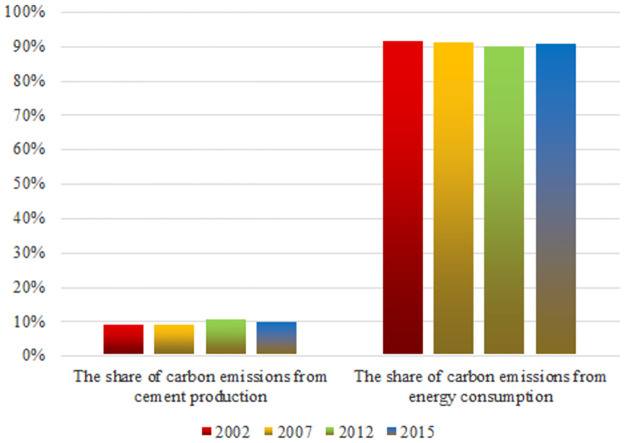
The share of cement production carbon emissions and energy consumption carbon emissions.

Based on the total cement production data of China for 2002, 2007, 2012 and 2015 obtained in Table 6, the carbon emissions from China’s cement production can be calculated. Utilizing the energy consumption method, the carbon emissions generated by China’s energy use in 2002, 2007, 2012 and 2015 are estimated. The detailed calculation results of carbon emissions and energy-related carbon emissions from cement production are shown in Table 7. The shares of carbon emissions from cement production and energy consumption in the total carbon emissions are as shown in Fig 1.

**Table 7:** The carbon emission from cement production and energy consumption.

Year	2002	2007	2012	2015
The carbon emission from cement production(10 kilo-tons)	36402.4	68336.1	109658.9	117993.9
The carbon emission from energy consumption(10 kilo-tons)	379235.4	697518.0	962080.4	1130198.1

Note: Due to a large amount of data, only 1 digit after the decimal point was included.

As shown in Table 7 and Fig 1, the carbon emissions from the cement production process accounted for 8.76%, 8.92%, 10.23% and 9.45% of the total carbon emissions, in 2002, 2007, 2012 and 2015 respectively. This indicated that the proportion of carbon emissions from cement production showed a slight increase from 2002 to 2012, and a slight decline from 2012 to 2015. Overall, from 2002 to 2015, carbon emissions from cement production accounted for a relatively stable proportion of total carbon emissions, which was more than 8% each year.

The results can also be better explained. As the main raw material of real estate and national infrastructure construction, China’s cement production has maintained a high level from 2002 to 2015 [3], which leads to a large proportion of carbon emissions from cement production in the total carbon emissions that cannot be ignored. The above results are basically consistent with the results of Lin and Zhang (2016) [35], which indirectly supports the relative reliability of the results in this paper.

Therefore, in the calculation of China’s carbon emissions, the important factor of carbon emissions from cement production cannot be ignored. Since the cement production industry belongs to the non-metallic mineral sector, it is also necessary to consider the carbon emissions from cement production while measuring the carbon emissions efficiency in the non-metallic mineral industry.

### The analysis of cement production influencing the carbon emissions efficiency

Based on the input-output method, the carbon emissions generated by energy use in 42 sectors in China can be estimated. However, the 42 sectors in this paper have been merged into 9 industries. Therefore, the energy carbon emissions of the 42 sectors also need to be consolidated and combined to obtain energy-related carbon emissions from 9 industries. Among them, the cement production industry belongs to heavy industry. The calculation results of heavy industry carbon emissions are shown in Table 8 with absence and inclusion of carbon emissions from cement production.

**Table 8:** The annual carbon emissions in heavy industry.

Influencing factor	The carbon emission form heavy industry in 2002(10 kilo-tons)	The carbon emission form heavy industry in 2007(10 kilo-tons)	The carbon emission form heavy industry in 2012(10 kilo-tons)	The carbon emission form heavy industry in 2015(10 kilo-tons)
In the absence of carbon emissions from the cement production	71152.24614	172175.3138	209494.8042	215206.7616
After inclusion of emissions from the cement production	107554.6461	240511.4138	319153.7042	333200.6376

It is evident from Table 8 that if the carbon emissions from cement production were neglected, the heavy industry carbon emissions would be underestimated by 33.83%, 28.40%, 34.37%, and 35.41% in 2002, 2007, 2012, and 2015 respectively. Based on the above analysis, it is concluded that the carbon emissions from cement production has a large impact on the carbon emission measurement results of heavy industry, which cannot be ignored, while this factor has no impact on the carbon emission measurement results of other industries. Therefore, the subsequent carbon emissions efficiency measurement of heavy industry need to consider this factor, while the carbon emissions efficiency measurement of other industries does not need to consider this factor.

According to the China’s Input-Output Table for 2002, 2007, 2012 and 2015, the output of the 42 sectors in China can be obtained. Then, the output of the following 9 sectors can be obtained through consolidation. The detailed output results of the 9 sectors are shown in Table 9.

**Table 9:** The output of the combined nine major sectors in 2002–2015.

Sector	The output in 2002 (Ten thousand yuan)	The output in 2007 (Ten thousand yuan)	The output in 2012 (Ten thousand yuan)	The output in 2015 (Ten thousand yuan)
Agriculture	285787423	488929999.9	894213473	1070563649
Construction	281326817.3	627217352.1	1386125872	2019037211
Light Industry	462635993.6	1275951528	2162525697	2811598279
Transportation	140959758.1	317001112.9	619666563	814159179.1
Service	801967250.6	1606850027	3871061527	5610771713
Chemical Industry	215726168.2	619980925.8	1210245788	1562054667
Fossil Energy	137226095.6	413627941.3	779081771	754190692.7
Heavy Industry	729558607.9	2524170849	4606416553	5582147932
Power	79116902.6	314859883.7	486933591	589941821.6

**Fig 2:**
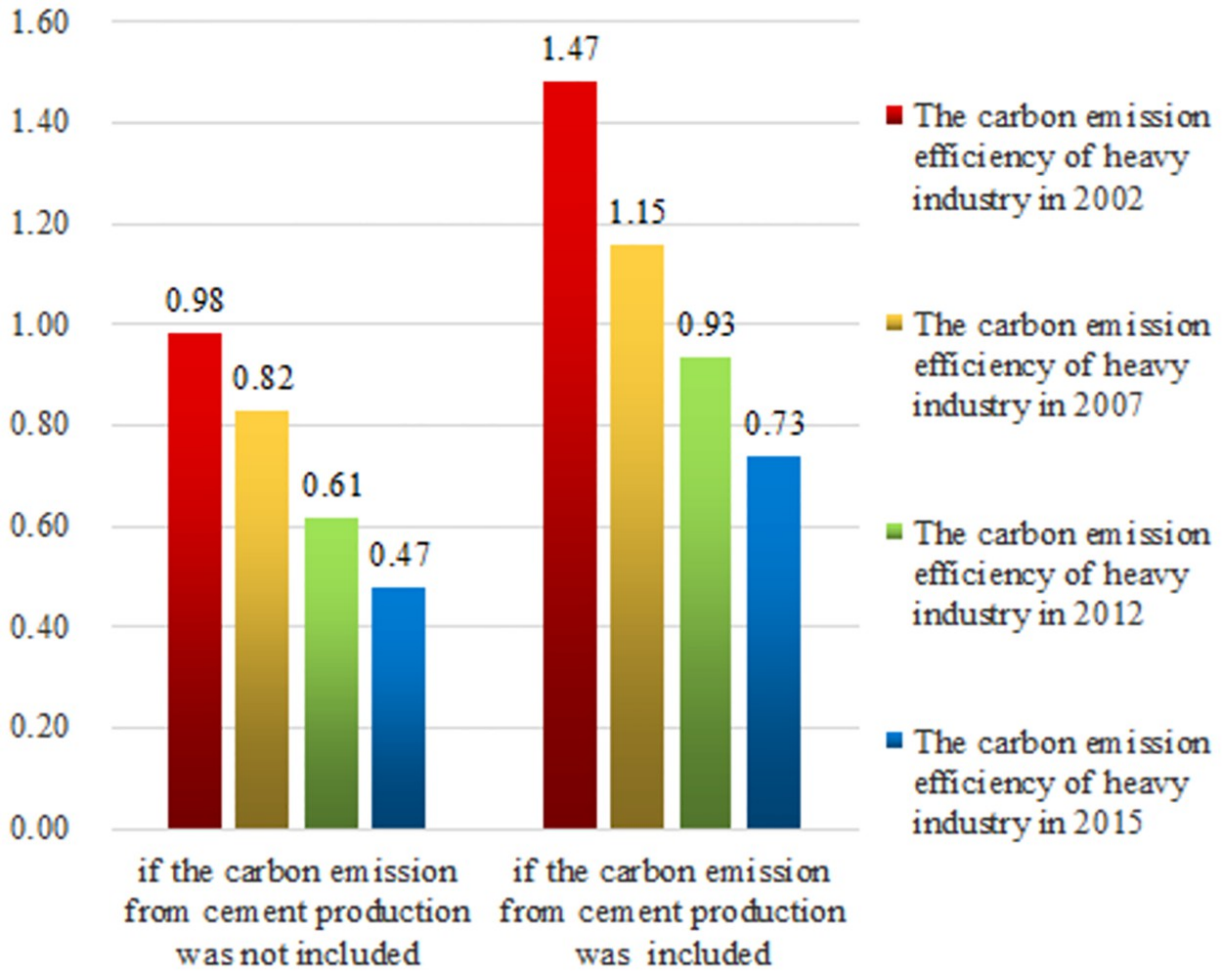
The carbon emissions efficiency in heavy industry.

Since the output of various sectors in Table 9 do not take into account the price conversion caused by the inflation, it is necessary to take the price for 2002 as the benchmark to convert the output for 2007, 2012 and 2015. Among them, the calculation results of carbon emissions efficiency of heavy industry in both situations (considering and not considering the carbon emission of cement production) are shown in Fig 2 (unit: ton / 10,000 yuan).

As is shown in Fig 2, if the carbon emissions from cement production are ignored, the results of heavy industry carbon emissions efficiency in 2002, 2007, 2012 and 2015 are 0.98, 0.82, 0.61 and 0.47. Whereas, if considering the carbon emission of cement production, the calculation results of heavy industry carbon emissions efficiency in 2002, 2007, 2012 and 2015 are 1.47, 1.15, 0.93 and 0.73 respectively. This is easy to explain. The cement industry is a heavy industry in the national economy. Due to China’s high demand for cement raw materials within the research range, the cement industry accounts for a large proportion of China’s heavy industry [36]. If the carbon emissions of heavy industry only consider the energy-related carbon emissions and ignore the carbon emission from cement production, it will inevitably lead to the actual calculation results of carbon emissions from heavy industry being underestimated, and the carbon emissions per unit output being also underestimated. Therefore, it can be concluded that the factor of carbon emission from cement production cannot be ignored for measuring the carbon emissions efficiency of heavy industry.

### The evolution of industrial carbon emissions efficiency

This paper mainly analyzes the evolution of industrial carbon emissions efficiency from 2002 to 2015 from two perspectives. On the one hand, we analyze the optimization of carbon emissions efficiency from the perspective of the entire industry system. On the other hand, we also analyze the differences in carbon emissions efficiency and the degree of optimization in each specific industry.

#### Analysis of the evolution of total carbon emissions efficiency of the industry

**Fig 3:**
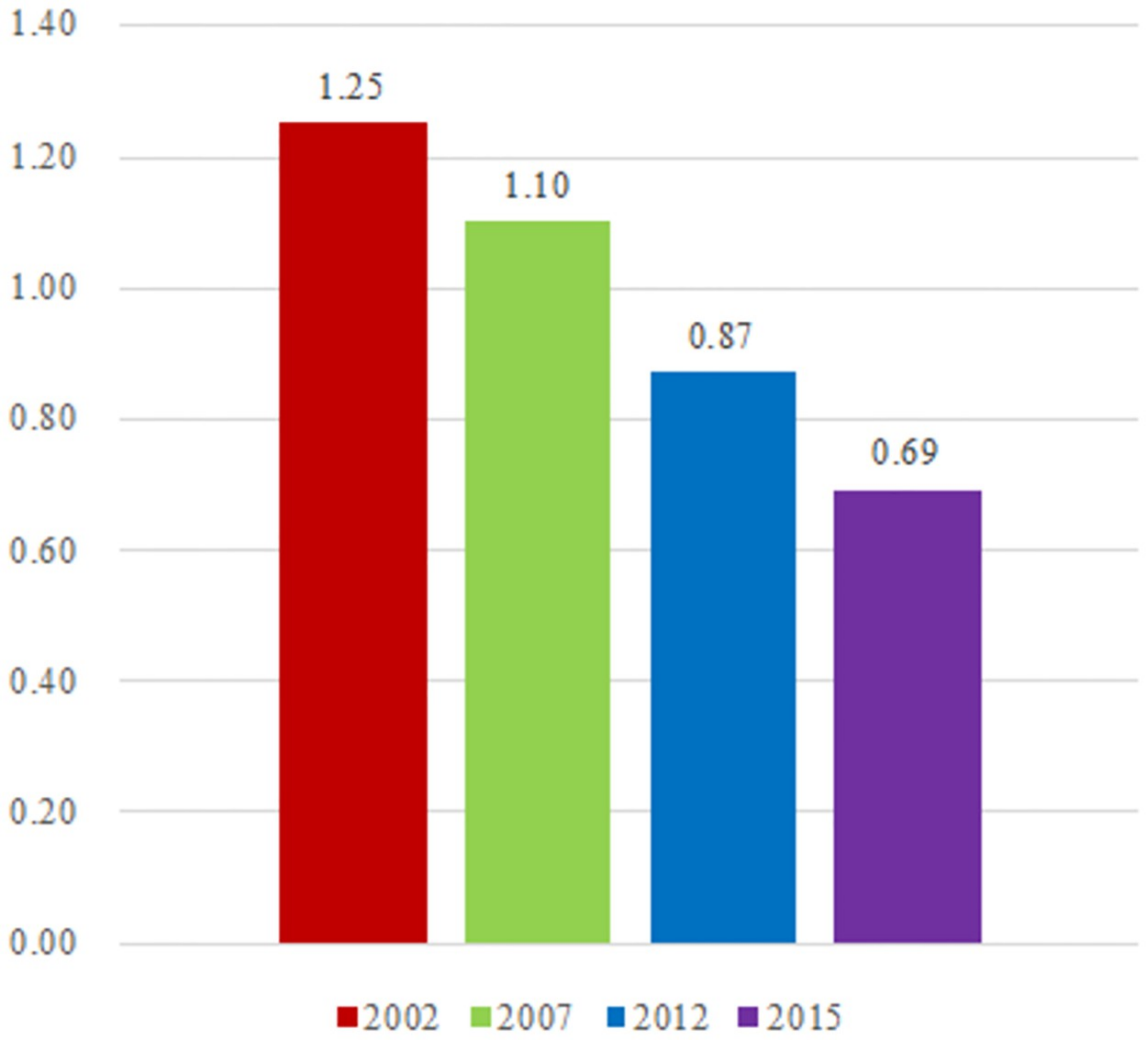
The evolution of industrial carbon emissions efficiency.

Based on the consideration of carbon emissions from cement production, China’s industrial carbon emissions efficiency in 2002, 2007, 2012 and 2015 was measured. The result is shown in Fig 3 (unit: ton / 10,000 yuan).

As shown in Fig 3, China’s industrial carbon emissions efficiency in 2002, 2007, 2012 and 2015 was 1.25, 1.10, 0.87 and 0.69. We can conclude that from 2002 to 2007, China’s industrial carbon emissions efficiency was optimized 1.13 times; from 2007 to 2012, China’s industrial carbon emissions efficiency was optimized 1.26 times; from 2012 to 2015, China’s industrial carbon emissions efficiency was optimized 1.26 times. This indicates that China’s industrial carbon emissions efficiency has been continuously optimized from 2002 to 2015 with a stable optimization degree. In general, compared with 2002, China’s industrial carbon emissions efficiency has increased by about two times in 2015. This is easy to explain. Since industrial carbon emissions are closely related to energy consumption, national industrial planning and policies, the national industrial planning of the Eleventh Five-year Plan and the Twelfth Five-year Plan within the research range can provide the possible explanations for the above phenomenon. In the Eleventh Five-year Plan, it is proposed to improve the resource utilization efficiency. More specifically, during the Eleventh Five-year Plan period, the energy consumption per unit of GDP will be reduced by about 20%, which is close to the 0.17 times increase in the carbon emissions efficiency of the industrial system from 2007 to 2010. Meanwhile, because the benchmark period of the eleventh five year plan is 2005 and the benchmark period of this paper is 2007, there is a certain quantitative gap that can be explained. In the Twelfth Five-year Plan, it is proposed to improve the resource utilization efficiency. More specifically, during the Twelfth Five-year Plan period, the energy consumption per unit of GDP will be reduced by about 16%, which is close to the 0.25 times increase in the carbon emissions efficiency of the industrial system from 2010 to 2015.

#### Analysis of the evolution of carbon emissions efficiency in various industries

Considering the carbon emission of cement production, the carbon emissions efficiency of various industries from 2002 to 2015 is estimated and shown in Table 10. The detailed results of the optimization of carbon emissions efficiency in various industries from 2002 to 2015 are shown in Table 11.

**Table 10:** The carbon emissions efficiency of various sectors (2002–2015).

Sector	2002	2007	2012	2015
Light Industry	0.3534	0.2481	0.1476	0.0753
Agriculture	0.3893	0.1131	0.1222	0.0689
Service	0.3989	0.1936	0.1168	0.0852
Construction	0.4418	0.2558	0.1035	0.0883
Heavy Industry	1.4742	1.1520	0.9312	0.7320
Chemical Industry	1.4986	1.4657	1.1514	1.0170
Transportation	2.1428	1.9905	1.3379	1.1501
Fossil Energy	3.1875	3.9717	3.7748	4.9555
Power	13.4357	6.6717	7.0138	5.2587

**Table 11:** The optimization of carbon emissions efficiency in various sectors (2002–2015).

Sector	2002–2007	2007–2012	2012–2015	2002–2015
Agriculture	3.44	0.93	1.77	5.65
Service	2.06	1.66	1.37	4.68
Power	2.01	0.95	1.33	2.55
Construction	1.73	2.47	1.17	5.00
Light Industry	1.42	1.68	1.96	4.69
Heavy Industry	1.28	1.24	1.27	2.01
Transportation	1.08	1.49	1.16	1.86
Chemical Industry	1.02	1.27	1.13	1.47
Fossil Energy	0.80	1.05	0.76	0.64

From the industrial structure analysis of Table 10, it can be concluded that from 2002 to 2015, the carbon emissions efficiency of various industries in China is generally different. Among them, agriculture, service, light industry and construction are generally sectors with higher carbon emissions efficiency, while power, fossil energy, transportation, chemical and heavy industry are generally sectors with low carbon emissions efficiency, especially low for the two sectors of power and fossil energy.

There are two reasons for the above phenomenon. From the analysis of industrial input factors, agriculture, service, light and construction industries are labor intensive industries, while power, fossil energy, transportation, chemical industry and heavy industry are energy input intensive industries. Besides this, the energy input of agriculture, service, light industry and construction are mainly based on clean energy such as power, while the power, fossil energy, transportation, chemical and heavy industries are mainly fossil based energy. For example, China’s power industry relies on coal-fired power generation, and the fossil energy sector relies on high-carbon energy sources such as coal. Therefore, for the energy conservation and emissions reduction in the future, China should pay attentions to improve the carbon emissions efficiency of the power sector, fossil energy, transportation, chemical and heavy industry, and thereby increase the proportion of low-carbon industry such as light industry, agriculture and service to improve industrial structure.

The optimization of carbon emissions efficiency in various sectors is illustrated in Table 11. It can be noted from Table 11 that the optimization of carbon emissions efficiency in agriculture, construction, light and service industry is increasing by 5.65, 5.00, 4.69 and 4.68 times respectively during 2002–2015. This is attributed to the fact that these industries belong to labor intensive industries with less energy dependent. Hence, the output of these industries can significantly be increased through mechanization and technological innovation.

Similarly, the optimization of carbon emissions efficiency in the power, heavy industry, transportation and chemical industries show an increase of 2.55, 2.01, 1.86 and 1.47 times respectively during 2002–2015. This is because these industries are both energy and capital intensive industries. These industries can improve industrial efficiency by introducing new technologies but with slower pace due to the large scale of operations, different from service industries which can update at a faster rate. For example, technical updates in the mechanical and power industries generally take more than five years. Therefore, the carbon emissions efficiency of these industries is optimized with insignificant optimization degree.

However, from 2002 to 2015, the fossil energy’s carbon emissions efficiency decreased by 0.36 times. This is because fossil energy is a typical energy intensive industry with a large renewal cost and a long cycle. In addition, the output of fossil energy is closely related to energy prices, and the prices of traditional fossil fuels such as coal showed a decline from 2011–2015. The above reasons lead to a very slow optimization of the carbon emissions efficiency of the fossil energy industry and even showing a negative growth. It can therefore be concluded that the Chinese government’s subsequent efforts to reduce emissions and improve carbon efficiency should focus on the power sector, chemical and fossil energy industry through appropriate policy guidance.

## Conclusion and policy recommendations

The excessive carbon emissions not only intensify the global climate change, but also seriously restrict the sustainable development of social economy. However, improving industrial carbon emissions efficiency is the most directly effective way to reduce emissions. Therefore, accurate measurement and analysis of carbon emissions efficiency and evolution characteristics of China’s industrial system is the basis for China to reduce emissions by improving carbon emissions efficiency. Based on this, this paper calculates and analyzes the industrial carbon emissions efficiency and evolution characteristics of China from 2002 to 2015 by using energy consumption method and input-output method. The results show that (1) If carbon emissions from cement production are ignored and only energy-related carbon emissions are considered, the calculation results of carbon emissions efficiency of heavy industry will be overestimated about 30%. (2) Compared with 2002, China’s industrial carbon emissions efficiency increased by about twice in 2015. Specifically, the optimization of carbon emissions efficiency in agriculture, construction, light industry and service industry is very obvious, which has increased by 5.65, 5.00, 4.69 and 4.68 times respectively; The optimization of carbon emissions efficiency in power sector, heavy industry, transportation and chemical industry was obvious, which increased by 2.55, 2.01, 1.86 and 1.47 times respectively; The carbon emissions efficiency of the fossil energy sector has decreased by 0.36 times. (3) The carbon emissions efficiency of various industries has significant differences. Among them, agriculture, service industry, light industry and construction industry are generally industries with higher carbon emissions efficiency, while power sector, fossil energy sector, transportation industry, chemical industry and heavy industry are generally industries with lower carbon emissions efficiency, especially power and fossil energy sectors.

Based on the above research conclusions, the following policy recommendations can be drawn. (1) We need to fully realize that the accuracy of carbon emission and carbon emissions efficiency calculation results is of great significance for measuring energy and environmental problems. Meanwhile, we also actively promote the research on the measurements of carbon emissions and carbon emissions efficiency, and improve the accuracy of calculation. The research results show that when calculating the carbon emissions efficiency of heavy industry in the industrial system, if the carbon emission of cement production is ignored, the carbon emissions efficiency will be overestimation more than 30%. Therefore, this factor needs to be considered when calculating the carbon emissions efficiency of heavy industry in the industrial system. But it does not need to be considered when calculating the carbon emissions efficiency of other industries. (2) China’s government needs to actively optimize the industrial structure and increasing the proportion of the structure serving low-carbon industries such as and light industry have a positive effect on carbon dioxide emission reduction. Meanwhile, the future optimization of industrial carbon emissions efficiency should focus on industries with low carbon emissions efficiency, such as power sector, fossil energy, transportation, chemical industry and heavy industry. There is still a lot of room for these industries to reduce emissions by optimizing carbon emissions efficiency. (3) For the energy industry, the optimization of carbon emissions efficiency is still not obvious, especially in the fossil energy sector. Therefore, in the process of follow-up industrial low-carbon development, we need to pay special attention to the low-carbon process of energy industry, actively promote the innovation and upgrading of energy industry technology, and improve the carbon emissions efficiency of energy industry in combination with corresponding policy guidance.

In addition, the future research gaps of this paper are as follows. (1) Because this paper uses the input-output analysis method to calculate the carbon emissions efficiency of industries on the demand-side, the research time of this paper can only be up to 2015, and the latest industrial carbon emissions efficiency cannot be calculated. (2) This paper uses input-output analysis method and energy consumption method to calculate China’s industrial carbon emissions efficiency, but does not further analyze the factors affecting China’s industrial carbon emissions efficiency, which is also the direction of follow-up research.

## Supporting information

S1 Appendix The sector classification. (DOCX)

S2 Appendix The abbreviations and their explanatory notes. (DOCX)
