# Supplementary material for: Analysis on carbon emissions efficiency differences and optimization evolution of China’s industrial system: An input-output analysis
Source: PLoS One. 2022 Mar 24;17(3):e0258147. doi: 10.1371/journal.pone.0258147 (PMC8947079; doi:10.1371/journal.pone.0258147)
Supplement: S1 Appendix — (DOCX) [file pone.0258147.s001.docx]

**Appendix Table** 1 The sector classification

| Sectors | Content |
| --- | --- |
| 1 Agriculture | Agriculture, Forestry, Animal Husbandry, and Fishery |
| 2 Fossil Energy | Coal Mining and Processing, Oil Mining and Processing, Natural Gas Mining and Processing, Production and Supply of Gas |
| 3 Power | Production and Supply of Power and Heating |
| 4 Heavy Industry | Metal Ores Mining and Processing, Non-metal Ores Mining and Processing, Other Mining Products, Non-metallic mineral products, Metal Smelting, Steel Rolling Processing, Metal Products, General Equipment Manufacturing, Special Equipment Manufacturing, Transportation Equipment Manufacturing, Electrical Machinery and Equipment Manufacturing, Telecommunications Equipment Manufacturing, Electronic Computer Manufacturing |
| 5 Light Industry | Foods and Alcoholic Beverages, Tobacco Products, Textile Materials Processing, Textile, Knitting Products Manufacturing, Textile, Clothing, Shoes, Hats Manufacturing, Leather, Fur, and Feathers and Its Products, Wood Processing and Furniture Manufacturing, Paper Making, Printing, Stationary and Sporting Goods Manufacturing, Instrument Manufacturing, Other Manufacturing Products, Waste Materials, Production and Supply of Water |
| 6 Chemical Industry | Chemical Products Manufacturing |
| 7 Construction | Construction |
| 8 Transportation | Transportation and Warehousing, Postal Service |
| 9 Service | Finance, Metal Products, Comprehensive Technical Service, Post and Telecommunications Information, Computer Services and Software Wholesale and Retail Trade, Accommodation and Catering Industry, Real Estate, Leasing and Business services, Research and Experimental Development Industry, Water Conservancy, Environment and Public Facilities Management, Residents Service and Other Services, Education, Health and Social Security, Culture, Sports and Entertainment, Public Administration and Social Organization |
