# Supplementary material for: Analysis on carbon emissions efficiency differences and optimization evolution of China’s industrial system: An input-output analysis
Source: PLoS One. 2022 Mar 24;17(3):e0258147. doi: 10.1371/journal.pone.0258147 (PMC8947079; doi:10.1371/journal.pone.0258147)
Supplement: S2 Appendix — (DOCX) [file pone.0258147.s002.docx]

**Appendix Table** 2 The abbreviations and their explanatory notes

| The abbreviations | The explanatory notes |
| --- | --- |
| APEC | The Asia-Pacific Economic Cooperation |
| IPCC | The Intergovernmental Panel on Climate Change |
| ISM | Industrial symbiosis measures |
| SBM | slacks-based measure |
| LPG | Liquefied petroleum gas |
| LNG | Liquefied natural Gas |
